# Effect of *Arthrospira (Spirulina) maxima* on Cadmium-Chloride-Induced Alterations in Sexual Behavior and Fertility in Male Wistar Rats

**DOI:** 10.3390/ph17030332

**Published:** 2024-03-03

**Authors:** Galván-Colorado Candelaria, García-Rodríguez Rosa Virginia, Mojica-Villegas María Angélica, García-Martínez Yuliana, Cristóbal-Luna José Melesio, Chamorro-Cevallos Germán

**Affiliations:** 1 Departamento de Farmacia, Escuela Nacional de Ciencias Biológicas, Instituto Politécnico Nacional, Av. Wilfrido Massieu 399, Mexico City C.P. 07738, Mexico; cgalvanc2001@alumno.ipn.mx (G.-C.C.); moviangel13@yahoo.com.mx (M.-V.M.A.); ygarciamart@hotmail.com (G.-M.Y.); josmcl@hotmail.com (C.-L.J.M.); 2 Instituto de Química Aplicada, Universidad Veracruzana, Luis Castelazo Ayala S/N Col. Industrial Ánimas, Xalapa-Enriquez, Veracruz C.P. 91190, Mexico

**Keywords:** cadmium, antioxidant, peroxidation, testosterone, spirulina, sexual behavior, sperm quality

## Abstract

Chronic exposure to potentially toxic elements (PTEs) such as cadmium (Cd) leads to male reproductive toxicity through the generation of oxidative stress. *Spirulina Arthrospira maxima* (AM) is a cyanobacterium that has been consumed since ancient times for its high nutritional value, and in recent years for its antiviral, hepatoprotective, hypoglycemic, anticancer, and antioxidant effects, among others. This study aimed to evaluate the effects of AM against the damage to reproductive health induced by Cd. A total of 48 10-week-old sexually experienced male Wistar rats were distributed in five groups (*n* = 8): control; vehicle (tween-water); cadmium chloride (CdCl_2_) 5 mg/kg; and three doses of AM (100, 200 and 400 mg/kg) + CdCl_2_ 5 mg/kg. All treatments were orally administered once a day for 36 consecutive days. At the end, sexual behavior was evaluated, and semen, testicle, and blood samples were obtained to analyze sperm quality, enzymatic activity, and testosterone levels, respectively. Rats exposed to Cd showed a decrease in sexual behavior, as well as in the quality of reproductive health, and an increase in oxidative stress; while rats exposed simultaneously to AM + Cd showed an improvement in all this parameters. Based on our results, we believe that the mechanism by which AM exerts its effect could be attributed to the presence of phycobiliproteins. These compounds are responsible for exerting an antioxidant effect and chelating effect on elements such as Cd.

## 1. Introduction

Due to certain industrial and anthropogenic activities, the availability and exposure of potentially toxic elements (PTEs) has increased in the last three to four decades, resulting in negative effects on human health from the contamination of water, air, and food. According to the World Health Organization (WHO), almost the entire population (99%) breathes air that endangers their health by exceeding the quality limits recommended by the WHO [1,2]. Most highly toxic PTEs, including cadmium (Cd), can frequently react with biological systems to form cations upon the loss of one or more electrons within vital macromolecules, causing serious damage to different organs of the body through both acute and chronic toxic effects [2]. One of the major impacts of this situation comprises the alteration of the endocrine system and the increase in the incidence of male infertility [3,4]. According to statistics, infertility affects 15% of couples worldwide, and about 40–50% of the causes are attributed to the male factor, since seminal quality tends to persistently decrease [5,6]. Exposure to PTEs is considered a risk factor associated with male infertility in 30% of infertile couples worldwide. Various authors have reported the effects of arsenic, Cd, lead, and mercury on male fertility, such as reduced sperm and hormone production and sperm quality [7,8].

Cd has different mechanisms of reproductive toxicity; unlike other metals, once absorbed into the body, Cd induces effects at relatively low doses. It has a long half-life at the biological level of between 20 and 40 years, accumulating in the body for considerable periods of time [6,9]. Acute and chronic exposure to Cd is associated with damage to the testicular vascular structure, impaired functionality, inflammation, apoptosis, Sertoli and Leydig cell cytotoxicity, and oxidative stress damage [10,11,12]. The EFSA (European Food Safety Authority) has established a provisional tolerable weekly dose (PTWI), representing the weekly amount of a substance that is considered safe for human health in the long term, which for Cd was 2.5 µm/kg. Meanwhile, the EPA (United States Environmental Protection Agency) has established a chronic exposure reference level (RfD), which is the daily amount of a substance that is expected to be safe for the general population, which for cadmium is 0.001 mg/kg/day [13].

The Fenton reaction is one of the reactions that derives oxidative stress by creating hydroxyl radicals from hydrogen peroxide, induced by Cd, and decreases the antioxidant activity of important endogenous enzymes, such as superoxide dismutase, glutathione peroxidase, catalase, glutathione reductase, and glutathione-S-transferase, increasing the production of reactive oxygen species [14,15].

Reactive oxygen species, on the other hand, have a high affinity for polyunsaturated fatty acids in the plasma membrane, damaging many cells, including spermatozoa [16]. Another way of inducing infertility through oxidative stress is the release of ROS through the hypothalamic axes of hormone release, acting as an endocrine disruptor. An increased intake of exogenous antioxidants would ameliorate the damage caused by oxidative stress, mainly derived from foods or medicinal plants, especially those containing polyphenols or carotenoids, conferring numerous biological effects [17].

Recently, antioxidants found in various natural products have gained significant relevance in preventing or reducing diseases whose etiology involves an increase in reactive oxygen species. Numerous investigations have demonstrated the substantial protective effect of consuming foods rich in antioxidants and sequestering agents against damage caused by oxidative stress. Therefore, it has been determined that by enhancing endogenous antioxidant defenses or by supplementing the diet with exogenous antioxidants, several chronic diseases can be prevented or their progression can be slowed [18,19]. Additionally, there is a growing trend of prescribing them to many patients with fertility problems due to their wide availability and proven safety. Consequently, they have been shown to improve key semen parameters, such as increasing motility, concentration, and viability, as well as the activation of endogenous antioxidants [20,21,22,23].

In this sense, microalgae have been the subject of multiple scientific investigations due to their diverse chemical, biological, and nutritional properties. Not to mention, they are a prominent natural source of promising compounds with pharmacological activity, as are several of their pigments [24]. Cyanobacteria are a large group of microorganisms belonging to microalgae. Although there are several species within the genus *Arthrospira*, the most studied species are *Spirulina platensis, Spirulina fusiformis*, and *Spirulina maxima* [25]. This last has gained great importance in numerous studies. This blue–green filamentous microorganism has a spiral morphology characterized by a marked antioxidant activity in vivo and in vitro. It has been described that it thrives in alkaline and warm environments, in both marine and fresh waters in Asia, Africa, Europe, and South and North America. *Spirulina Arthrospira maxima* (AM) was used as food by the Aztec civilization during the pre-Hispanic era [26].

Nowadays, it is consumed as a food supplement due to its interesting nutritional content and its multiple pharmacological properties. Its nutritional profile suggests an abundance of proteins (60–70% of its composition), lipids (7.2%), carbohydrates (10.3%), fiber (8.5%), and minerals such as iron, calcium, magnesium, potassium, zinc, and selenium (6.9%). Additionally, it is also an important source of essential amino acids, polyunsaturated fatty acids, and sterols. It is rich in vitamins, with important amounts of niacin B3, vitamin B6, B12, and vitamin K. It also contains pigments such as carotene, chlorophyll A, and phycocyanin [26,27,28].

It has also been shown to offer numerous biological activities, including antiviral [29], antimicrobial [30], antitumor [31], immunomodulatory [32], antiallergic [31], and antihypertensive properties [33,34]. Several studies have confirmed the important anti-inflammatory activity [35], associated in part with effects on the modulation of the intestinal microbiota through various signaling pathways, not yet defined, but which suppress the growth of pathogenic bacteria. Additionally, there is an antioxidant effect [36,37] exerted by the phycobiliprotein C-phycocyanin, which is the most abundant in AM.

**Figure 1:**
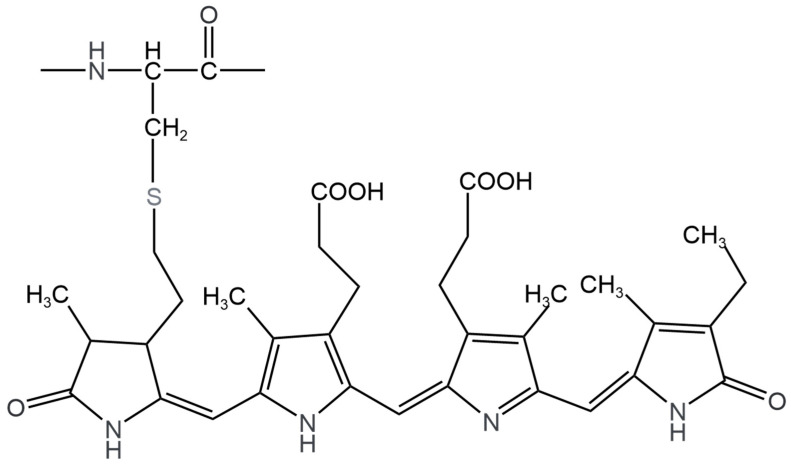
Structure of phycocyanobilin, the chromophore group of phycobiliproteins responsible for antioxidant activity [40].

Structurally, C-phycocyanin (CPC) is composed of phycocyanobilin as a chromophore (Figure 1). The CPC monomer is formed by different α and β polypeptide units and, in turn, covalently linked to one or two phycocyanobilins. In previous studies carried out in our laboratory, it has been possible to obtain an aqueous extract of phycobiliproteins from AM, using freeze–thaw techniques, obtaining yields of between 30 and 38%, highlighting the abundant presence of CPC, as well as allophycocyanin and phycoerythrobilin in smaller amounts [38,39]. Montaño-González [39] reported the protective effect of phycobiliproteins on sperm quality and oxidative stress, finding improvement in some parameters; however, so far, there are no reports on the antioxidant protective effect exerted by AM on the male reproductive system. Therefore, the main objective of this study is to evaluate the effect of AM on sexual performance, sperm quality, and oxidative stress caused by Cd in the reproductive system of male Wistar rats, which could have a synergistic effect due to the activity exerted by its components together.

## 2. Results

### 2.1. Sexual Behavior

**Figure 2:**
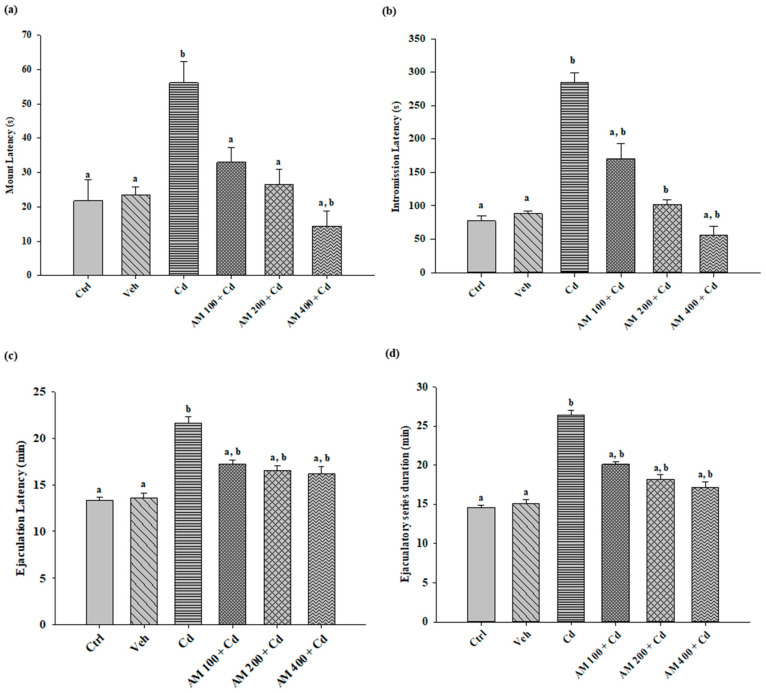
Latency periods in sexual behavior: (**a**) riding latency; (**b**) latency of intromission; (**c**) ejaculation latency; (**d**) duration of the ejaculatory series. Ctrl, control; Veh, vehicle; Cd, cadmium; AM, *Arthrospira maxima.* Values are represented as mean ± SEM, *n* = 8. Versus CdCl_2_; a = *p* < 0.05, versus control; b = *p* < 0.05.

The results for mating, intromission, and ejaculation latency times, as well as the duration of the ejaculatory series (Figure 2), show that the administration of AM decreased the latency times in all evaluated parameters for the animals that received AM 400 + Cd, reaching a behavior similar to that of the control group in all tests, with a dose-dependent effect. The group treated with Cd, which exhibited a significant increase in the recorded times for each test, consequently demonstrated significant differences compared to the treated groups and the control group.

### 2.2. Semen Quality

**Figure 3:**
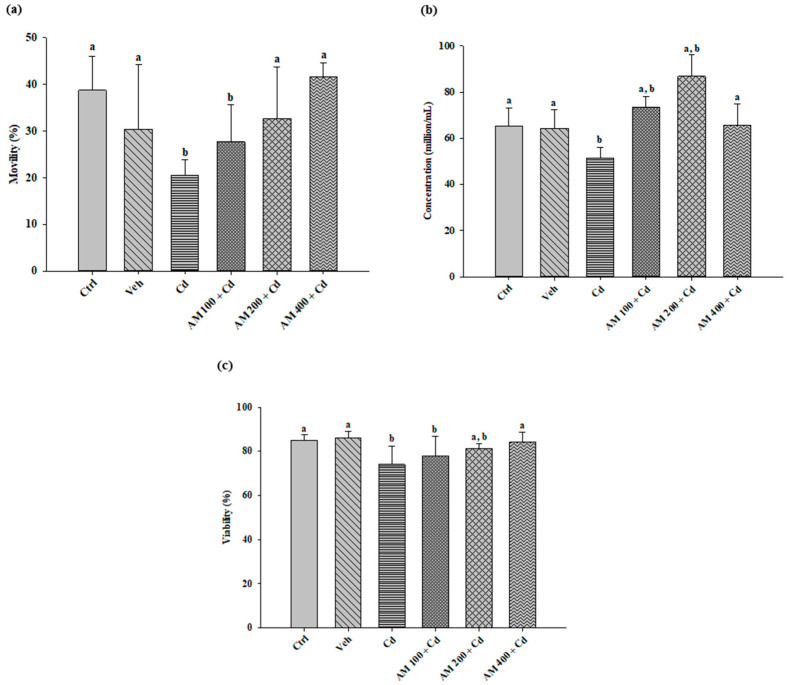
Semen quality. (**a**) Progressive mobility; (**b**) concentration; (**c**) viability. Ctrl, control; Veh, vehicle; Cd, cadmium; AM, *Spirulina maxima.* Values are represented as mean ± SEM, *n* = 8. Versus CdCl_2_; a = *p* < 0.05, versus control; b = *p* < 0.05.

Analysis of sperm quality (Figure 3) revealed that Cd administration led to a significant decrease in sperm motility (20.58%) compared to the control group (38.71%). In contrast, the groups treated with AM exhibited a dose-dependent effect, with a noticeable recovery in motility percentage.

Similarly, the Cd group exhibited a significant decrease in the number of spermatozoa, recording 51.38 million/mL compared to the control group. In contrast, the rats administered doses of 100, 200, and 400 mg/kg of AM as treatment showed a significant increase in sperm concentration (73.38, 86.88, and 65.50 million/mL, respectively) compared to the results of the Cd group.

Viability analysis revealed a sperm viability of 85.31% in healthy animals. In contrast, a decrease was observed in the Cd group (74.06%), which showed a slight recovery after the administration of AM at doses of 100, 200, and 400 mg/kg (78.06%, 81.28%, and 84.27%, respectively), indicating a dose-dependent effect.

### 2.3. Biochemical Assays

**Figure 4:**
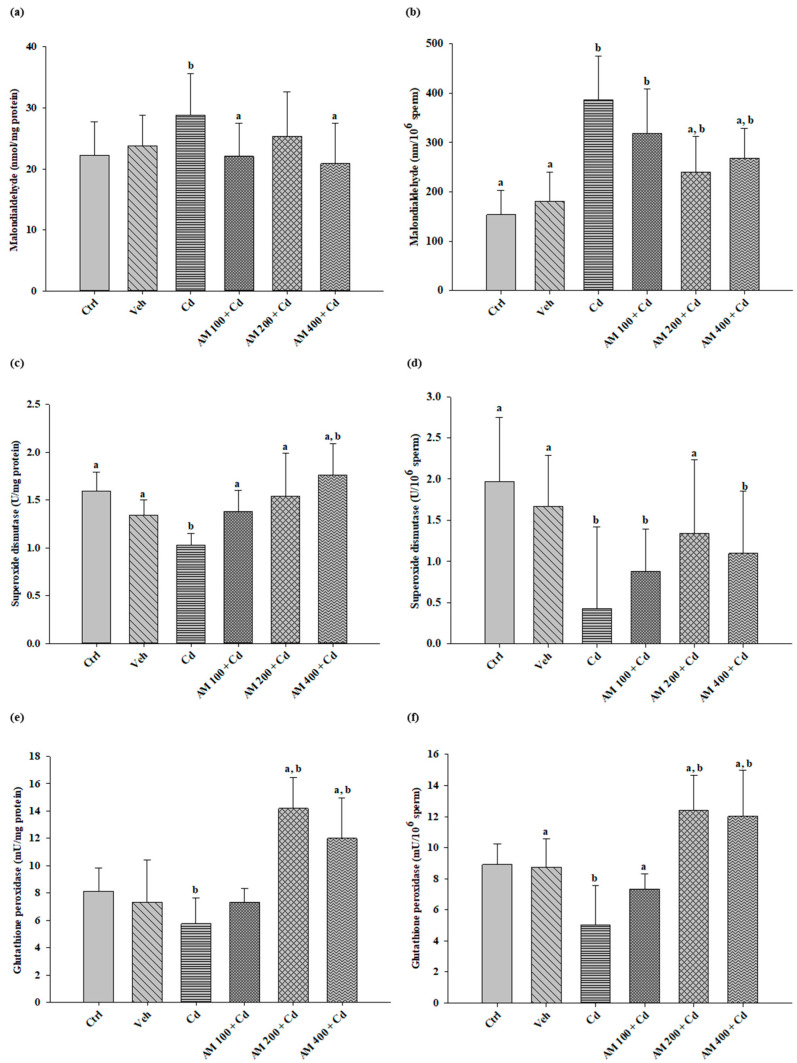
Biochemical analyses: (**a**) lipoperoxidation in the testis; (**b**) lipoperoxidation in sperm cells; (**c**) SOD levels in the testis; (**d**) SOD levels in sperm cells; (**e**) Gpx levels in the testis; (**f**) Gpx levels in sperm cells. Ctrl, control; Veh, vehicle; Cd, cadmium; AM, *Spiruline maxima.* Values are represented as mean ± SEM, *n* = 8. Versus CdCl_2_; a = *p* < 0.05, versus control; b = *p* < 0.05.

Lipoperoxidation in the testicular tissue (Figure 4a) for the Cd group showed an increase of 29.82% compared to the control group. Significant differences were observed in the groups administered with AM at doses of 100 and 400 mg/kg, indicating a decrease in MDA production of 23% and 27%, respectively. In spermatozoa (Figure 4b), lipoperoxidation increased (387 nmol MDA) compared to the control group (153 nmol MDA). However, in the groups treated with AM at doses of 200 and 400 mg/kg, MDA levels decreased by up to 38% and 30%, respectively.

In the testicular tissue (Figure 4c) of the Cd group, SOD decreased considerably (38%) compared to the control group. However, in the groups treated with AM, SOD increased in a dose-dependent manner. In sperm cells (Figure 4d), SOD also decreased by up to 78% in the Cd group compared to the control group. Significant differences were found in the groups treated with AM, showing a potentiation of SOD activity in the animals treated at a dose of 200 mg/kg of SM.

Regarding Gpx in the testis (Figure 4e), Cd intoxication induced a decrease of up to 29% in Gpx activity in rats. Treatment with AM at doses of 200 and 400 mg/kg increased Gpx activity by 60% compared to the control group. In the analysis of Gpx in spermatozoa (Figure 4f), a decrease was also observed in the Cd group (43%) compared to the control group. Treatment with AM improved enzyme activity, especially at doses of 200 and 400 mg/kg, increasing it by up to 50% compared to the control group.

**Figure 5:**
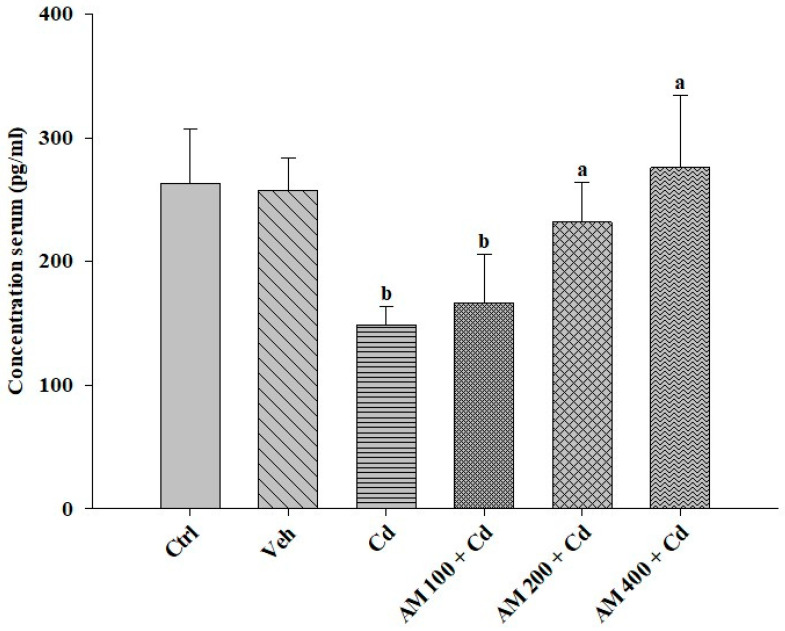
Graph showing the results of the evaluation of testosterone levels in the different groups of rats. Ctrl, control; Veh, vehicle; Cd, cadmium; SM, *Spiruline maxima.* Values are represented as mean ± SEM, *n* = 8. Versus CdCl_2_; a = *p* < 0.05, versus control; b = *p* < 0.05.

The serum testosterone concentration (Figure 5) was adversely affected in the Cd group, decreasing by up to 44% compared to the control group. In contrast, in the SM-treated rats, there was a rebound of 85% and 56% at doses of 200 and 400 mg/kg, respectively, compared to the Cd-administered group.

## 3. Discussion

Currently, the population is exposed to various environmental factors that promote damage to reproductive health. The application of nutraceuticals and antioxidant compounds represents a promising strategy to prevent and counteract the toxicity of potentially toxic elements (PTEs) in various organs. These agents target different molecular pathways, such as activating the NrF2 pathway to restore redox balance, activating inflammasomes, and/or chelating elements like Cd. This approach offers a comprehensive and promising defense against the detrimental effects of environmental factors on reproductive health [41].

Several studies have emphasized the induction of oxidative stress as the primary mechanism leading to the deterioration of sperm cells and, consequently, fertility, due to PTEs. As a result, the consumption of natural antioxidants has been suggested as a potential alternative. In this context, AM has shown various benefits for the treatment of various ailments in both experimental animals and patient studies [26,31,33,36,37]. Moreover, it has demonstrated its lack of toxicity in both short- and long-term studies [26].

In the present investigation, we evaluated the impact of AM on the damage to sexual behavior caused by Cd. FAO/WHO has established a safe intake guideline known as the Provisional Tolerable Weekly Intake (PTWI) for Cd, which is 400–500 μg per person per week or 140–160 μg/day [40,42]; in daily scenarios, it is very difficult for humans to be exposed to such high concentrations of Cd as 5 mg/kg, because chronic exposure occurs at lower doses for prolonged periods [13], so the choice of the dose used in our experiment was limited for this reason, which could act as a limitation in terms of direct application to alleviate Cd intoxication. Nevertheless, the dose used and reported in previous research [43,44,45] has allowed us to generate effective damage at the testicular level in the animal model, and at the same time, it has great scientific value because it presents AM as an alternative to prevent or reduce the damage from the substances of this type to which we are exposed on a daily basis. It is important to mention that cadmium tends to bioaccumulate [40] and, therefore, it is possible to observe possible protective effects from certain antioxidant products.

Regarding the results obtained, an increase in latency time was observed in the groups treated with Cd, suggesting a potential alteration in endocrine regulation. Cd has been shown to influence the biosynthesis of hormones, thereby affecting sexual behavior [35].

In contrast, the decrease in mounting, intromission, and ejaculation latencies observed in rats treated with AM at doses of 200 and 400 mg/kg reflects a positive response in the appetitive sexual behavior of male rats. This effect of AM is presumed to be related to the presence of amino acids such as L-arginine, which can be enzymatically synthesized and increase the concentration of nitric oxide, directly influencing the erection mechanism. Additionally, vitamin E, a key element in the production of sex hormones, such as testosterone, and phycobiliproteins, with their chelating effect on free radicals produced by PTEs, contribute to mitigating damage and reactivating androgen biosynthesis [24]. It is noteworthy that the decrease in latency times observed in the group treated with 400 mg/kg of AM may not always be beneficial, as very low latency can translate into an increase in cardiac output due to excess energy. The physical activity of sexual intercourse is associated with an increased myocardial oxygen demand and the heightened activation of the sympathetic nervous system, which can lead to myocardial ischemia [38,46].

The alterations and decrease in sperm quality parameters observed in the group of rats administered with Cd were similar to those shown in several previous studies [47,48,49]. According to the description by Adamkovikova et al. [50] and upon comparing the obtained data, it is likely that in rats exposed to Cd, the synthesis of epidermal proteins and other substances associated with sperm maturation were affected, triggering structural and biochemical changes. This is especially notable because both the testicular membrane and spermatozoa are highly susceptible to reactive oxygen species (ROS) attacks, attributed to their composition being rich in polyunsaturated fatty acids [43,50,51].

The protective antioxidant system of semen comprises both enzymatic and non-enzymatic factors. Vitamin complexes, glutathione, carotenoids, coenzyme Q10, carnitine, minerals, and more are highly efficient antioxidants that safeguard against oxidative damage. Consequently, trials have been conducted to evaluate the beneficial effects of these antioxidants on sperm quality parameters in men with idiopathic male fertility issues. These studies have shown improvements in these parameters, leading to an increase in fertility rates [50]. AM is a supplement that integrates many of these antioxidants, including vitamins, minerals such as zinc, and carotenoids [24]. As a result, treatment with AM has demonstrated an improvement in sperm quality. It has been observed that Cd decreases the concentration of zinc in testicular tissue, which acts as a cofactor for SOD. From this, it can be speculated that AM, through its antioxidant activity conferred by phycocyanin, decreases the Cd load in testicular tissue and restores the depleted zinc, thereby exerting a protective effect on sperm cells [24,52,53].

The Increase in MDA in the Cd group is associated with cell membrane impairment, mitochondrial dysfunction, and endogenous enzyme inhibition, leading to the accumulation of free radicals such as superoxide anion (O_2_^−^), hydroxyl radical (OH^−^), and hydrogen peroxide (H_2_O_2_) production. Both the testicular tissue and sperm membrane contain high concentrations of polyunsaturated fatty acids, making them highly susceptible to peroxidative damage [54,55]. In contrast, natural antioxidants such as vitamin E, resveratrol, and curcumin provide protection to the testis from reactive oxygen species (ROS) damage [50]. Therefore, it is presumed that AM (200 and 400 mg/kg) improved peroxidation levels due to the effect of phycobiliproteins, which favor the activation of endogenous antioxidants (SOD, CAT, Gpx, etc.), promoting protective effects.

It has been documented that the phycobiliproteins present in species such as AM exert a chelating effect that inactivates heavy metal ions, inhibiting the formation of peroxides and hydroperoxides, which are responsible for accelerating the process of lipoperoxidation in fatty acids [56,57]. Inside the cells, phycocyanobilin is reduced to phycocyanorubin, sharing the ability of bilirubin to inhibit NADPH oxidase complexes, thus suppressing the activation of inflammasomes triggered by superoxide production [58]. Similar results were reported by Bashandy et al. [54] for the work in which they evaluated the ability of AM as a treatment for arsenic-induced reproductive toxicity.

Regarding the evaluation of the endogenous antioxidant defense system, this study first observed that Cd increased reactive oxygen species (ROS) in testicular tissue by decreasing zinc usage through competitive binding. Consequently, this led to a significant reduction in superoxide dismutase (SOD) activity [55]. In sperm cells, a similar phenomenon occurs; Cd, due to its oxidation state similarity, can compete with Cu at the site of the membrane and cytoplasmic proteins. Copper, along with zinc and manganese, acts as a cofactor for SOD. Therefore, the imbalance of essential minerals or a decrease in their concentration is associated with reduced SOD activity, leading to increased oxidative stress and consequent enzymatic saturation [59,60].

Gpx is a selenium-dependent enzyme that uses reduced glutathione (GSH) as a reducing agent. Our results show that the decrease in Gpx could be related to the affinity of Cd to thiol groups in proteins, so it also includes the binding to cysteine in GSH resulting in the inactivation of Gpx; the binding of Cd to sulfhydryl groups can decrease functional GSH, affecting the structure and function of GSH-dependent anti-oxidant enzymes [61]. This coincides with what has been reported by various authors, such as Adi et al. [55]. In contrast, studies where researchers have tested the effect of zinc, selenium, and copper, as well as vitamin E, to mitigate the effects of PTEs such as Cd, have achieved a considerable increase in endogenous enzyme activity, similar to that reported in this study, in rats supplemented with AM (doses 200 and 400 mg/kg) [54]. These results can be justified due to the integration of components, such as vitamin C, vitamin E, manganese [53,62], Se, Zn, Cu, and Mg, that confer a good performance in endogenous antioxidant defense [24,53].

According to the results we obtained for serum testosterone quantification, we observed a decrease related to the previously exposed poor seminal quality, which aligns with findings reported by other authors, such as Bashandy et al. [54], Obembe and Raji [56], and Mouro et al. [63]. These studies suggest that the decrease in testosterone is linked to mitochondrial dysfunction in Leydig cells, a crucial source of testicular testosterone influenced by follicle-stimulating hormone (FSH) and luteinizing hormone (LH), which are, in turn, controlled by GnRH produced in the hypothalamus [63,64]. In contrast, the recovery of testosterone levels in the groups treated with AM could be influenced by the antioxidant activity of phycobiliproteins present in it. As mentioned, these activate endogenous antioxidant enzymes, protecting Leydig cells, similar to the observed effects in other Spirulina species, like AM [53,57]. In a study by Babaknejad et al. [65], zinc (Zn) and magnesium (Mg) were tested as a treatment for the adverse effects of Cd, resulting in decreased oxidative stress and increased testosterone levels. Zn is an essential antioxidant in free radical scavenging enzymes such as Cu/Zn-SOD, acting as an inhibitor of NADPH oxidase and preventing free radical generation by interfering with chemical group interactions with iron. Notably, AM also contains these trace elements in its composition, potentially contributing to the observed increase in testosterone concentration.

Based on the above, the analysis suggests that nutraceuticals offer the potential to harness the beneficial properties of natural compounds present in food to counteract the toxic effects of Cd, while also promoting the adoption of healthy dietary habits. Additionally, good agricultural practices, such as monitoring soil quality, play a crucial role, as soil contamination by Cd often results from the use of Cd-rich fertilizers. It is important not to overlook the translational perspective, ensuring that findings from basic research can be applied to clinical practice and integrated into daily life [66].

## 4. Materials and Methods

The AM used in this study originated from the Atacama Desert in northern Chile, where the cultivation and production plant of AM (Solarium Biotechnology & AEH, Iquique, Chile) is located. This plant is owned by the company Alimentos Esenciales para la Humanidad, S.A de C.V. based in Mexico City, Mexico, which generously donated a sample from production batch SDX-9902. The product holds Kosher Certification, ensuring the purity of the food for human consumption, and is available in the form of a fine blue–green powder.

### 4.1. Experimental Animals

Wistar rats (200–250 g) were procured from the breeding colony of the Universidad Autónoma Metropolitana (UAM), Unidad Xochimilco, located in Mexico City, Mexico. A total of 48 male Wistar rats and 20 ovariectomized female Wistar rats were employed as stimuli for the study. The animals were housed in cages with a raised floor and wide mesh (to prevent coprophagia) within a dedicated animal room under standard temperature conditions (22 ± 1 °C) and a 12 h light/dark cycle. They received a standard diet and access to purified water ad libitum throughout the experiment. The rats were acclimated to the laboratory environment for 15 days before the initiation of the experimental protocols. Following each experiment, the animals were euthanized using a carbon dioxide euthanasia chamber.

All procedures and animal handling were conducted in strict adherence to the Mexican Official Standard (NOM ZOO-062-200-1999) [67] titled “Technical Specifications for the Production, Care, and Use of Laboratory Animals”. This was carried out in accordance with the Bioethics Committee of the National School of Biological Sciences of the National Polytechnic Institute, under the guidelines of ZOO-021-2019, Mexico City. Additionally, the procedures followed the ARRIVE guidelines.

### 4.2. Preparation of Females for Mating, Selection of Animals, and Preparation for Experiments

Males exhibiting the best sexual performance were selected for inclusion in the study. The evaluation was conducted in round acrylic cages measuring 60 cm in diameter and 40 cm in height, with a bed of sawdust. Each male rat was paired with a receptive female, allowing free interaction between them, and latency times were recorded. This training was repeated in triplicate for each male on different days and with different females to prevent sexual exhaustion. Healthy males capable of completing the ejaculatory series in less than 15 min and across three consecutive copulation sessions were considered for the study. Males that did not reach this target were withdrawn from the study [35].

As for the females used as stimuli, they underwent bilateral ovariectomy prior to the study. Anesthesia was induced with an intraperitoneal (i.p.) dose of sodium pentobarbital (60 mg/kg), and both ovaries were removed, followed by ligation of the oviducts. Subsequently, the ovariectomized females were allowed a 15-day recovery period after surgery [68]. Prior to sexual behavior assays, these ovariectomized females were subcutaneously (s.c.) administered estradiol benzoate (12 µg/kg) (Sigma Chemical Co., Ltd., Schnelldorf, Germany) 24 h before initiating sexual behavior experiments and progesterone (3 mg/kg) (Sigma Chemical Co., Ltd.) 4 h before initiating sexual experiments. This hormonal treatment was administered to induce estrus.

### 4.3. Treatment and Experimental Groups

The animals were divided into the following groups (*n* = 8). Group I was the control group (untreated healthy rats). Group II received water–tween 9:1 (vehicle). Group III was induced toxicity by administering CdCl_2_ (5 mg/kg); while groups IV, V, and VI received CdCl_2_ (5 mg/kg) and, 60 min later, AM (doses: 100, 200, and 400 mg/kg, respectively). The administration was conducted via the intragastric route using water as a vehicle, once daily for 36 consecutive days. To demonstrate the protective effect of AM, a dose of Cd was sought that would lead to deterioration in sexual behavior, testicular damage, and oxidative stress. According to previously published studies, the doses of Cd used in standardized models range from 5 to 15 mg/kg; the dose of 5 mg/kg was chosen because it is the lowest dose of Cd that has been shown to produce significant damage [43,44,45,69].

### 4.4. Sexual Performance Evaluation

At the end of the administration period, each male rat was paired with a receptive female. The sexual behavior of male rats was observed from one side of the cage to record sexual behavioral parameters for a duration of 30 min. The tests were conducted between 16:00 and 19:00 h under red light conditions, following the procedure outlined by Yakubu and Akanji [70]. From this observation, the latency times (mounting, intromission, ejaculation), and the duration of the ejaculatory series were recorded.

### 4.5. Sample Collection

At the end of the sexual behavior experiments, the rats were sacrificed via cervical dislocation. Blood samples were collected via cardiac puncture, from which serum was obtained; semen was collected from the vas deferens, and testicular tissue was obtained.

### 4.6. Sperm Quality Analysis

#### 4.6.1. Evaluation of Sperm Motility and Sperm Count

Ten µL of fresh semen were placed on a slide, and 200 spermatozoa were analyzed under a 40× objective lens. Each cell was classified based on its individual motility (fast, slow, and immobile progressive). The result was obtained by summing the spermatozoa with fast and slow progressive motility, expressed as a percentage. The sperm count was determined using a hemocytometer by placing 10 µL of the sperm suspension, diluted in 5% formaldehyde, on each of the two counting chambers and observing with a 40× objective. The result is expressed as million/mL [43].

#### 4.6.2. Evaluation of Sperm Viability

Eosin/nigrosin staining was employed, relying on the principle that dead cells with damaged plasma membranes permit the entry of the dye. The sample and dye were applied to a slide, with the sample spread in a thin layer, allowed to air dry, and subsequently examined under 100× microscopy. Two hundred spermatozoa were considered and classified as live or dead. The results are expressed as a percentage.

### 4.7. Biochemical Analyses

Biochemical analyses were performed on sperm cells (5 million spermatozoa/200 µL SSF) and on testicular tissue (50 mg of sonicated testicular tissue in 500 µL SSF).

#### 4.7.1. Lipid Peroxidation

Lipid peroxidation was determined via the reaction of reactive substances with thiobarbituric acid [71]; malondialdehyde (MDA) is produced as a final result of lipid peroxidation and reacts with thiobarbituric acid forming a TBA-MDA complex, which is analyzed via spectrophotometry. The results were expressed as nmol of MDA.

#### 4.7.2. Superoxide Dismutase

Superoxide dismutase was estimated using the commercial Ransod kit (RANDOX^®^ Laboratories Ltd., Crumlin, UK) employing xanthine and xanthine oxidase to generate superoxide radicals that react with 2-(4-iodophenyl)-3-(4-nitrophenol)-5-phenyltetrazolium chloride to form a red formazan dye. The superoxide dismutase activity was measured by the degree of inhibition of this reaction determined via spectrophotometry, and the result is expressed in U/mg protein [72].

#### 4.7.3. Glutathione Peroxidase

Glutathione peroxidase was evaluated using the Ransel diagnostic kit (RANDOX^®^ Laboratories Ltd., UK) following the manufacturer’s instructions. Gpx catalyzes the oxidation of glutathione by cumene hydroperoxide. In the presence of glutathione reductase and NADPH, oxidized glutathione is immediately converted to the reduced form with the concomitant oxidation of NADPH to NADP^+^. Two absorbance readings were performed for each sample, and the results are expressed as UGpx/mg protein [73].

### 4.8. Determination of Testosterone Concentration

The testosterone concentration was determined via enzyme-linked immunoadsorption competitive immunoassay. For this purpose, the commercial ELISA kit (testosterone EIA Kit Cayman Chemical Company) was used, following the manufacturer’s specifications. The plates were read in an ELISA reader at 415 nm. The results are expressed as pg/mL.

### 4.9. Statistical Analysis

Data were analyzed by testing and meeting the assumptions of homogeneity of variance, then analyzed via one-way ANOVA analysis of variance and Dunnet’s post hoc tests; significant differences were considered significant when *p* < 0.05. Values are expressed as mean ± SEM (*n* = 8). Both the analysis and the figures were performed using Sigma Plot software version 12.0.

## 5. Conclusions

Exposure to Cd induces damage to reproductive health, disrupting sexual performance and spermatogenesis by elevating oxidative stress and reducing testosterone levels. The administration of AM effectively reversed the damage caused by Cd, demonstrating a dose–response effect in most cases. This reversal was evident in improved sexual performance, the protection of spermatozoa, reduced lipoperoxidation, the restoration of endogenous antioxidant system activity, and elevated testosterone levels. The proposed protective mechanism of AM is attributed to its potent antioxidant activity. Additionally, a parallel mechanism not assessed in the present investigation may be associated with the presence of Zn, Se, vitamin E, and phycobiliproteins in AM, potentially fostering a synergistic effect. This, in addition to its antioxidant activity, could contribute to other mechanisms, further countering the oxidative effects of Cd.

## Data Availability

Data are contained within the article.
